# Tactile Signatures and Hand Motion Intent Recognition for Wearable Assistive Devices

**DOI:** 10.3389/frobt.2019.00124

**Published:** 2019-11-21

**Authors:** Thekla Stefanou, Greg Chance, Tareq Assaf, Sanja Dogramadzi

**Affiliations:** ^1^ZITI, Heidelberg University, Heidelberg, Germany; ^2^Bristol Robotics Laboratory, Department of Computer Science, University of Bristol, Bristol, United Kingdom; ^3^Department of Electronic and Electrical Engineering, University of Bath, Bath, United Kingdom; ^4^Bristol Robotics Laboratory, Department of Engineering Design and Mathematics, University of the West England, Bristol, United Kingdom

**Keywords:** motion intent, wearable sensors, upper-limb, tactile sensing, assistive devices

## Abstract

Within the field of robotics and autonomous systems where there is a human in the loop, intent recognition plays an important role. This is especially true for wearable assistive devices used for rehabilitation, particularly post-stroke recovery. This paper reports results on the use of tactile patterns to detect weak muscle contractions in the forearm while at the same time associating these patterns with the muscle synergies during different grips. To investigate this concept, a series of experiments with healthy participants were carried out using a tactile arm brace (TAB) on the forearm while performing four different types of grip. The expected force patterns were established by analysing the muscle synergies of the four grip types and the forearm physiology. The results showed that the tactile signatures of the forearm recorded on the TAB align with the anticipated force patterns. Furthermore, a linear separability of the data across all four grip types was identified. Using the TAB data, machine learning algorithms achieved a 99% classification accuracy. The TAB results were highly comparable to a similar commercial intent recognition system based on a surface electromyography (sEMG) sensing.

## 1. Introduction

The motivation behind this work lies in empowering individuals with mobility impairments to rehabilitate after stroke or similar debilitating conditions. With an aging population (World Health Organisation, 2014), keeping people active and independent for as long as possible is becoming increasingly important. The number of occupational therapists and physiotherapists in the UK is not sufficient to cover the needs of this aging demographic (McHugh and Swain, 2013). Rehabilitation robots have shown a potential to alleviate this problem by assisting in controlled, repetitive movements typically provided by the therapists. By recognizing patients' motion intent, the wearable rehabilitative devices can further assist in performing the desired movement. These devices should provide just enough force to move the limbs as intended keeping the patient in the control loop (Warraich and Kleim, 2010).

Intent recognition has been the subject of numerous studies and various sensing modalities have been used over the years. The consistency and accuracy of motion intent recognition devices varies though depending on the conditions (patient strength, skin moisture etc.). Muscle contraction gives rise to two types of signals, electrical and mechanical. The former, in the form of electromyography (EMG) has been implemented in many commercial products for motion intent recognition (Thalmic Labs, 2015b; Wearable Devices Ltd., 2019). Most recent research has shifted toward the observation of the mechanical signals produced during muscle contraction, namely mechanomyography (MMG) and force myography (FMG), also referred to as tactile imaging. Over the last few decades EMG motion intent recognition has been widely implemented in assisted living (Kiguchi and Hayashi, 2012), rehabilitation (Rehab-Robotics Company Limited, 2017) and prosthetic systems (Atzori et al., 2014). Nonetheless, EMG controlled systems have still not reached acceptable, consistent performance as indicated by (Farina et al., 2014). One of the main inconsistency factors is that the electric potential detected on the surface of the skin as a result of muscle contraction could be affected by skin impedance, while adipose tissue can induce crosstalk. Despite the acknowledged limitations of EMG devices the electromyography device market is expected to grow in the years to come (Technavio, 2018). This shows that there is a demand for understanding and measuring muscle activity that can be incorporated in motion intent recognition systems and integrated into wearable rehabilitation devices.

More recent works report results of the integration of EMG sensors with other means of sensing such as force sensing (Guo et al., 2015; McIntosh et al., 2016). Motion intent recognition studies have observed the mechanical signals produced as a result of the contraction of the muscles (Yap et al., 2016). Two different approaches have been implemented; MMG and FMG. MMG detects low frequency muscle vibrations and their velocity and intensity, usually through the use of accelerometers (Islam et al., 2013). FMG observes muscle architectural changes during contraction that can be monitored by force or stiffness changes on the skin surface (Phillips and Craelius, 2017). A plethora of research on the use of MMG considers the characterization of muscle activity and fatigue as well as the diagnosis of neuromuscular disorders (Islam et al., 2013), sometimes in combination with sEMG (Tarata, 2009). More recent research has focused on using MMG as a control input for prosthesis and medical rehabilitation devices (Ding et al., 2017a,b). In Ding et al. (2017b) the MMG based system, which used an inertial measurement unit, with an accelerometer and a gyroscope, achieved a 94% accuracy when distinguishing between the fingers performing tapping motions.

The idea that the volumetric and shape changes that take place inside the muscle can be monitored on the skin surface and used as an indication of motion intent was first captured by Moromugi et al. (2004). They implemented push buttons with load sensors indented in the skin to capture “muscle stiffness” for the purpose of actuating a prosthetic hand. Wininger et al. (2008) performed one of the first studies implementing FSR sensing to predict grip force in hand prostheses. Using a grip dynamometer, they mapped the readings measured during gripping and the pressure exerted by the forearm on the force measuring cuff. After testing the concept on healthy young adults, they concluded that this is a useful alternative to EMG. Furthermore, a high resolution tactile sensor system developed by Schürmann was used in a proof of concept study to create tactile images of the anterior forearm (Castellini and Koiva, 2013). It was later shown that pressure sensing is not only a cheaper alternative to sEMG but it also provides better measurement consistency (Ravindra and Castellini, 2014). The same sensory technology was embedded and tested in a tactile sensor bracelet (Koiva et al., 2015). A feasibility study on FMG technology was performed by Cho et al. (2016) which resulted in a classification accuracy of over 70%, while Xiao and Menon (2017) proved its robustness during on-the-fly verification. However, to the authors' knowledge, the majority of such systems have been used in high muscle engagement conditions which did not demonstrate the ability to differentiate subtle variations involved in different hand poses or during low-strength gripping. Furthermore, existing motion intent recognition systems rely heavily on machine learning (Yu and Lee, 2015) for data classification, which lacks transparency in the decision making process. Consequently, the control of these wearable devices can hinder user safety. This paper aims to fill the identified gaps in the body of knowledge toward further development of wearable, upper-limb, stroke rehabilitation devices.

A tactile arm brace (TAB) design and testing was performed and reported in Stefanou et al. (2018b), where common tactile patterns were identified with healthy participants. The questions we set to answer in this paper include:

- What is the performance of tactile sensing as a means of understanding motion intent under low strength conditions? How does it compare with the current approaches?
- How do tactile features relate to the muscle physiology? Are these features common within a population? Could a more transparent decision making system be developed?

Section 2 gives an overview of our user study and describes the grip types used and their muscle synergy analyses. The results of this study are presented in section 3 which compares the forearm's tactile signatures to its expected physiological states and employs machine learning techniques to classify the state of the hand. Section 4 compares the TAB performance to the Myo armband (Thalmic Labs, 2015c) based on sEMG.

## 2. Methodology

One of the main aims of this study was to establish the potential of the TAB in distinguishing between different hand motions produced by different combinations of muscle engagement. This section introduces the experimental set-up and procedure during this participant study followed up with analyses of the muscle contractions associated with the grips performed.

### 2.1. The Experimental Set-Up

**Figure 1:**
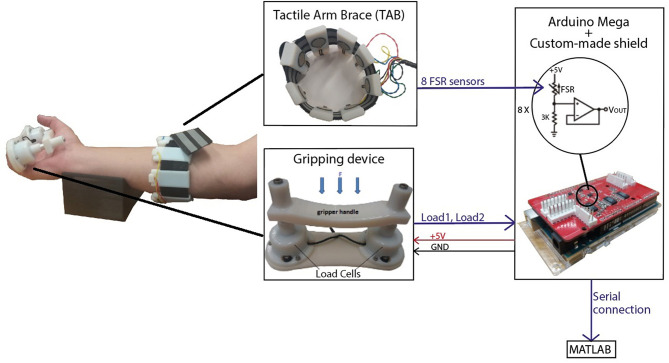
The experimental set-up included the tactile arm brace (TAB), the gripping device and a forearm support. An Arduino MEGA with a custom-made shield was used to capture the sensor data and transfer it to MATLAB in real-time.

The TAB is a low-cost sensorised arm brace (Figure 1). It consists of an armband fitted with 8 force sensitive resistive (FSR) sensors uniformly distributed around the user's forearm. Its purpose is to monitor the normal interaction forces, as detailed in Stefanou et al. (2018b), capturing tactile signatures of the forearm.

A gripping device was developed for these experiments. It comprises two load cells under a handle (Figure 1) (Stefanou et al., 2018a) and has a resolution of 0.27N and sensitivity of 0.17N. An average error of 1.79% was calculated in the range of 0N and 9.81N. The acquisition of the TAB and gripping device data was done using an Arduino MEGA on which a custom made board was mounted to provide 16-bit analog inputs with a sampling rate of 89Hz. Calibration of the TAB FSR sensors was performed prior to the experiments where the force on each TAB sensor was recorded as a function of time. The two load cells were also calibrated using a set of known weights. The communication between the Arduino, where all sensor data were captured, and the computer was synchronous.

### 2.2. Participant Study

Experiments were performed with 20 healthy participants, 10 male and 10 female. Ethics approval from the University of the West of England Ethics Committee was acquired as well as informed written consent from all participants. The TAB sensing surfaces were placed three quarters of the way up the length of the forearm.

#### 2.2.1. Experimental Procedure

**Figure 2:**
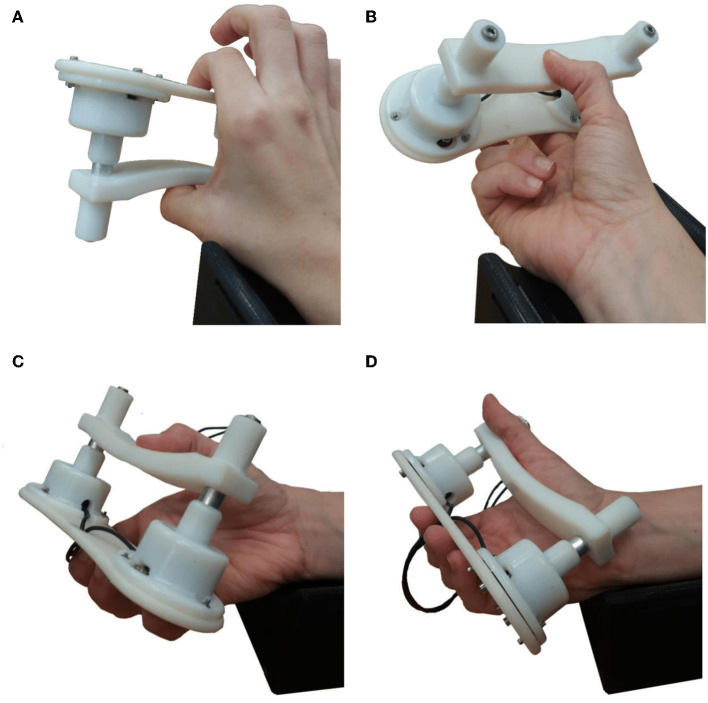
Photographs of the four grip configuration used with the gripping device. During the experiments the forearm always rested on the arm support shown in the supination position. **(A)** *Grip1*, the prismatic precision grip with two fingers. **(B)** *Grip2*, the prismatic precision grip with three fingers. **(C)** *Grip3*, the prismatic precision grip with five fingers. **(D)** *Grip4*, the prismatic power grip with five fingers.

Four different types of static grips were chosen (Figure 2), representative of grips typically used in activities of daily living and based on the feasibility and comfort of the gripping device. These included four prismatic grips; three precision grips, with 2 (Figure 2A), 3 (Figure 2B) and five fingers (Figure 2C), and a power grip using all 5 fingers (Figure 2D). The forearm was placed on the support in the supination position as shown in Figure 1.

The participants were given visual instructions on a monitor and verbal guidance during trials. Their seat height was adjusted accordingly so that they could comfortably place their forearm on the 3D-printed support shown in Figure 2. The experiment was repeated four times, once for each grip type, where each type of grip was performed 5 times. To create a variability in the sensor waveform profiles, the participants were instructed when to start gripping but were allowed to choose how quickly to grip and when/how quickly to let go. The instructions were given as follows:

- *GRIP* (*t*); initiate gripping
- *PREPARE* (*t+7*); warning to release the grip, if they have not done so already
- *3s* allowed for rest
- *GRIP* (*t+10*).

### 2.3. Grips and Muscle Synergy

This section analyses the four grip types shown in Figure 2. The activity of the forearm muscles during each grip type determines the tactile signatures expected to be recorded by the TAB. The contribution of each forearm muscle to each grip type was determined based on its anatomical and physiological parameters. The hand configuration, the placement of the fingers, the wrist angle and the supination/pronation of the forearm were taken into account when analysing the TAB sensor readings and the features that can distinguish the *griping* and *relaxed* states and the four grip types.

Previous studies have indicated that individual finger force contributions during 5-finger precision (trapezoid) gripping are proportional to each finger's strength (Radwin et al., 1992). The first three gripping configurations used are precision grips. The fourth grip type used, Figure 2D, is a power grip, with the thumb adducted. All four grip types have a prismatic shape and they were all performed with the forearm in the supination position resting on a 3D-printed support. The grips were selected according to the detailed taxonomy of human grasps presented by Feix et al. (2016). Table 1 details these four grips and the percentage force contribution of each finger as found in the studies by Radwin et al. (1992) and Kinoshita et al. (1996)^1^. The grip force limits used during the experiment for each grip type are presented in Table 1.

**Table 1:** The four grips performed during the experiment, the respective forces achieved with each one and the individual digit contributions (Radwin et al., 1992; Kinoshita et al., 1995, 1996), as well as the maximum force limits the participants in this study were instructed to not exceed.

		**Contribution (%)**						**Trials: max force**	
**GRIP**	**Grip type**	**I**	**M**	**R**	**L**	**Thumb**	**Strength**	**Force**	**% of**
						**position**	**max. (N)**		**strength**
*Grip1*	Precision,	100	N/A	N/A	N/A	Abducted	152.2	0.5 kg/4.9N	3.2
	prismatic								
*Grip2*	Precision	43	57	N/A	N/A	Abducted	121.8	1.0 kg/9.8N	8.0
	prismatic								
*Grip3*	Precision	35	26	20	19	Abducted	100	1.5 kg/14.7N	15
	prismatic								
*Grip4*	Power	25	35	25	14	Adducted	402	2.0 kg/19.6N	4.9
	prismatic								

**Figure 3:**
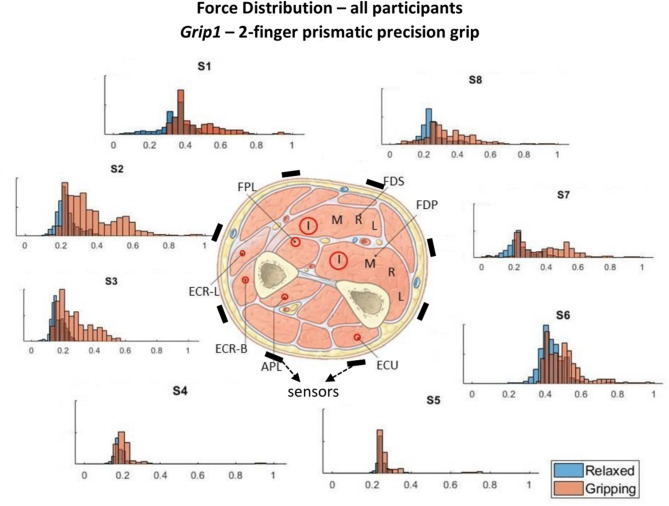
These histograms present the force distributions detected by each TAB sensor when performing *Grip1*, the 2-finger precision grip. The blue histograms present the TAB forces when the hand is *relaxed* and the orange when it's *gripping*. The approximate sensor locations are also indicated.

The pose and involvement of each hand digit and the wrist in each grip type determine the expected magnitude of the forearm muscle contractions. The contribution of each muscle and its proximity to individual TAB sensors, with any damping effects caused by the soft tissue, determine the expected tactile signature of the forearm. A detailed diagram of the forearm cross-section anatomy, just below the elbow, was used to associate each TAB sensor with the muscles in its proximity (Figure 3, each sensor annotated as S1-S8). The digit flexor muscles, the flexor digitorum superficialis (FDS) and flexor digitorum profundus (FDP) (Nordin and Frankel, 2001), flex the four fingers and the diagram provides details of their correspondences to the TAB sensors. The cross-section area of each muscle shown in Figure 3 has a direct correlation to its strength capacity (Cutts et al., 1991). The muscle compartments that control the middle finger joints are expected to produce the highest forces followed by the ones that control the index and ring fingers. This is important as each of the four parts of the FDP and FDS muscles has different proximities to the TAB sensors, and is thus expected to produce a different tactile signature. The interosseous membrane (Skahen et al., 1997) that divides the forearm into the anterior and posterior compartments is tensed when the forearm is in the supination position (McGinley and Kozin, 2001). This leads to effective damping that confines any muscle contraction effects (in particular in the inner forearm layer) to the compartment of its origin. This especially affects non-superficial muscle contraction effects on the TAB sensors.

#### 2.3.1. Individual Grip Type Analysis

During *Grip4* (Figure 2D), the thumb is adducted, which means that the extensor pollicis longus muscle is active. This does not happen with the three precision grips. The digit flexors have a high activity, from highest to lowest in order, these are the FDS, the FDP and the flexor pollicis longus (FPL). Furthermore, the wrist extensor muscles engage to a smaller extent as they contract to tighten the digit flexor tendons. Given the nature of the grip and the wrist orientation, the ulnar wrist extensor is expected to play a bigger role during *Grip4* than the radial wrist extensor. In contrast to *Grip4* (the power grip), in *Grip1* (Figure 2A), *Grip2* (Figure 2B) and *Grip3* (Figure 2C) the thumb is abducted and the ulnar deviation of the wrist is not as prominent. The hand configuration in *Grip4* engages the FDS more than in the other grips as there is a higher PIP (proximal interphalangeal) joint flexion (Nordin and Frankel, 2001). The DIP (distal interphalangeal) joint flexion is lesser though in this configuration compared to the others implying lower FDP engagement. Therefore, differences between the two are expected to be seen on the ventral radial part of the forearm. During *Grip1* and *Grip2* only some parts of the FDS and FDP muscles are active, flexing the index and middle fingers. The middle finger has a higher force contribution than the index in *Grip2* (Table 1), the 3-finger precision grip. It is therefore assumed that the parts of the muscles responsible for the actuation of the middle finger produce a greater contraction and hence a larger force on the TAB sensors in its proximity. The thumb abduction, actuated by the abductor pollicis longus (APL), is expected to be greater in *Grip3* than *Grip2* in order to position the thumb in opposition to both the index and middle fingers.

The expected sensor responses for each grip type are presented in Table 2; they are classified as Low/Medium/High.

**Table 2:** Expected muscle contraction and TAB sensor responses for the four different grips, *Grip1, Grip2, Grip3*, and *Grip4* (Figure 2).

	**Grip1**		**Grip2**	
**TAB sensor**	**Muscle activation**	**Expected change**	**Muscle activation**	**Expected change**
S1	FDS(I),FPL	Medium	FDS(I,M),FPL	Medium
S2	ECR-longus, FPL	Low-Medium	ECR-longus, FPL	Low-Medium
S3	ECR-brevis	Medium	ECR-brevis	Medium
S4	APL	Low-Medium	APL	Low-Medium
S5	ECU	Medium	ECU	Medium
S6	ECU	Low	FDP(M)	Low
S7	FDP(I)	Low	FDP(I,M)	Low-Medium
S8	FDS(I)	Medium	FDS(I,M)	High
	**Grip3**		**Grip4**	
**TAB sensor**	**Muscle activation**	**Expected change**	**Muscle activation**	**Expected change**
S1	FDS(I,M),FPL	Medium	FDS(I,M),FPL	Low-Medium
S2	ECR-longus, FPL	Medium	ECR-longus, FPL	Low-Medium
S3	ECR-brevis	Medium	ECR-brevis	Medium
S4	APL	Low-Medium	APL	Low
S5	ECU	Medium	ECU	Low
S6	FDP(M,R,L)	Medium	FDP(R,L)	High
S7	FDP(M,R), FDS(L)	Medium-High	FDP(M,R), FDS(L)	Medium-High
S8	FDP(I,M), FDS(I,M,R,L)	Medium-High	FDP(I,M), FDS(I,M,R,L)	High

### 2.4. Data Labeling

To label the data with the state of the hand for each grip type, *gripping* or *relaxed*, the onsets and terminations of gripping were determined using the gripping force measurements. An algorithm was developed to determine gripping instances by checking whether the grip force threshold of 0.49N has been exceeded and whether any proximal grip/release events have occurred. A release event is identified only after a grip event and when the grip force is <0.69N. Another criterion for determining the release event is whether at the midpoint between the possible release point and the corresponding grip point the grip force is higher than a certain threshold (which varies between the grip types). All thresholds used in the algorithm were based on the sensitivity and accuracy of the gripping device and determined by trial and error. Each data point was labeled with a number that indicates the configuration of the hand (one of four grip types presented in Figure 2) and whether the hand was *gripping* or *relaxed*.

## 3. Results and Analysis

The TAB sensor readings were scaled across all grip types using the minmax normalization technique between 0 and 1, for each participant individually. While the hand adopts each of the four different finger configurations without exerting any force on the gripping device, the tactile signature on the TAB changes. However, the tactile signatures have variations across the participants, i.e., the *relaxed* signature of one user is different to the *relaxed* signature of another user. This is due to the participants' different forearm sizes and shapes, TAB placement and fit, as well as the muscle to adipose tissue ratios. All these factors that affect the signature on the TAB, as would be expected, are especially evident when the hand is in one of the *relaxed* states. Not only does the force magnitude of the detected changes vary, but also the prominence of some force changes at certain parts of the forearm is higher than at others in different participants across hand configurations.

### 3.1. Tactile Signatures—A First Glance

**Figure 4:**
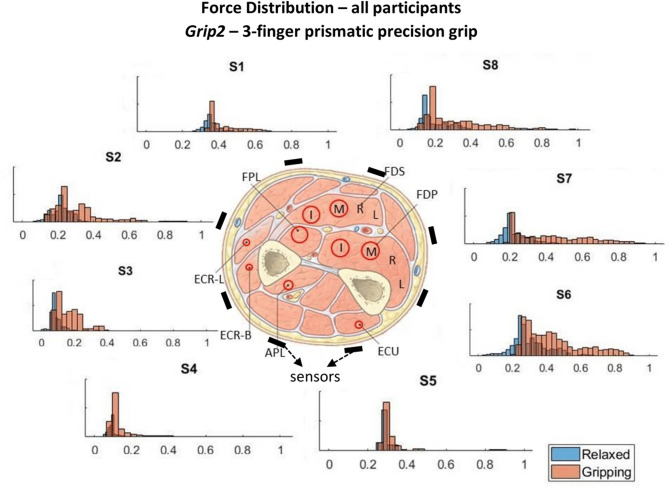
These histograms present the force distributions detected by each TAB sensor when performing *Grip2*, the 3-finger precision grip. The blue histograms present the TAB forces when the hand is *relaxed* and the orange when it's *gripping*. The approximate sensor locations are also indicated.

**Figure 5:**
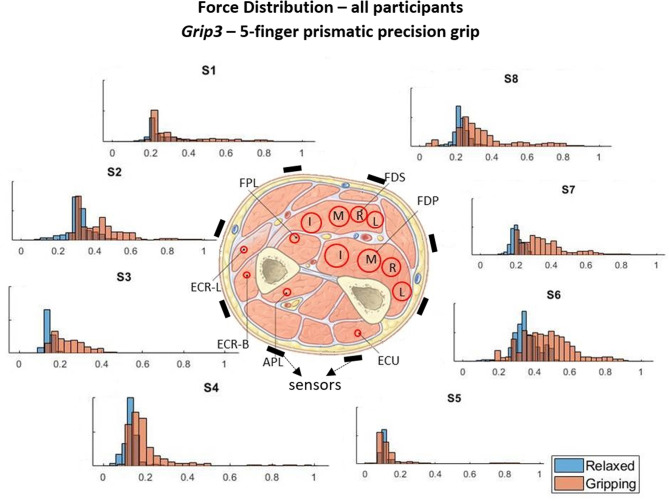
These histograms present the force distributions detected by each TAB sensor when performing *Grip3*, the 5-finger precision grip. The blue histograms present the TAB forces when the hand is *relaxed* and the orange when it's *gripping*. The approximate sensor locations are also indicated.

**Figure 6:**
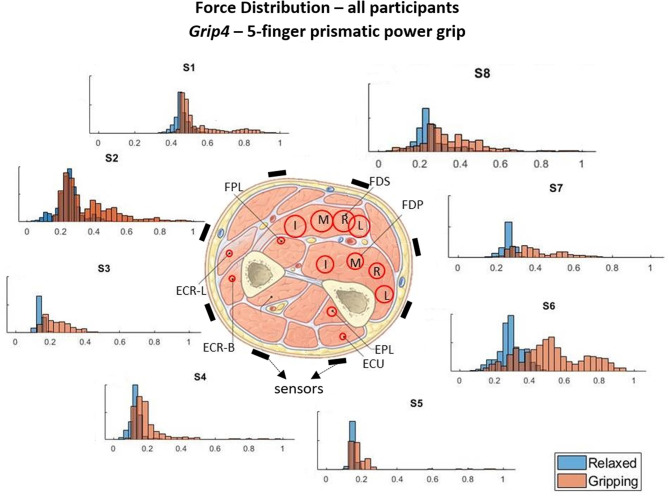
These histograms present the force distributions detected by each TAB sensor when performing *Grip4*, the 5-finger power grip. The blue histograms present the TAB forces when the hand is *relaxed* and the orange when it's *gripping*. The approximate sensor locations are also indicated.

**Figure 7:**
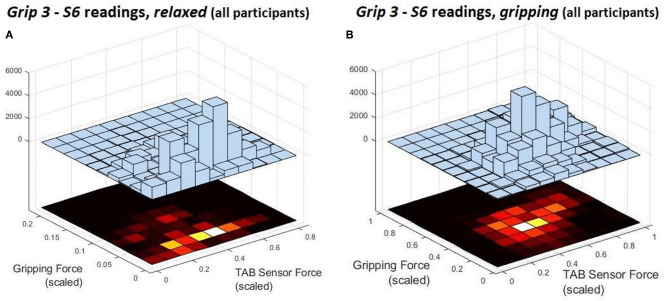
These are the deconstruction of a single histogram from Figure 5. They present the force distribution of the *S6* data against the gripping force recorded during *Grip3* when the hand is **(A)** *relaxed* and **(B)** *gripping*.

The force measurements around the forearm in the *gripping* and *relaxed* states for all participants are visualized in Figures 3–6, one for each grip type. At first glance, it is obvious that the data are not normally distributed and that generalizing for all participants may not be feasible. In order to further break down the data distribution and gain a better understanding of the results, bivariate histograms were produced (Figure 7). These visualize the frequency density of the individual sensor readings with respect to the grip force for each grip type.

Figures 3–6 show how the muscles engage during each grip type (indicated by red circles) and the response of each of the TAB sensors when the hand is *gripping* (orange histograms) and *relaxed* (blue histograms). The force frequency distribution of some sensors is significantly different in the *relaxed* and *gripping* states for particular grip types. For example, there is a clear distinction between the *relaxed* and *gripping* force distributions of *S6* for *Grip2, Grip3*, and *Grip4*. A very strong bi-modal distribution is visible for *Grip4* (Figure 6) while a clear shift of the median can be seen in *Grip3* (Figure 5). *Grip1* and *Grip2*, that differ only by the use of the middle finger have very similar tactile signatures when *gripping*, with the exception of the TAB sensors situated on the volar/ulnar side of the forearm; ie. *S7* and *S8*. The distributions have longer tails and higher frequencies, around 0.8 which can be explained by the parts of the FDS and FDP muscles that flex the middle finger (annotated with “M” in the forearm cross-section diagrams), which are located in the proximity of those two sensors. This is one example that demonstrates that despite variations across participants, the results do agree with the physiological analysis performed and expected tactile signatures as tabulated in Table 2. It was expected that *Grip1* and *Grip2* would cause similar forces on all TAB sensors with the exception of *S7* and *S8*. Comparisons with *Grip3* where the remaining parts of the FDP and FDS muscles, located in the inner/ulnar part of the forearm, are engaged as well, indicate that not only are the *S7* and *S8* responses higher but also that *S6*, located at the ulnar side of the forearm, is sensitive to *Grip3* as expected (Table 2). Overall, the results agree with the analysis performed and the expected outcomes.

Figure 7 shows the *S6* readings against the grip force during *Grip3* (scaled as indicated earlier). S6 was chosen because of its location in a region of the forearm where prominent force changes take place. This can be also seen in the histogram of *S6* in Figure 5 (*Grip 3*). The *relaxed* state data are presented in Figure 7A and the *gripping* data in Figure 7B. While *relaxed*, the recorded grip force mainly ranges between 0 and 5% of the maximum recorded. There is no correlation between the force measured by *S6* and the one measured by the gripping device. The variability of the forces recorded when the hand is *relaxed* could be due to the variability of TAB tightness on the participants' forearm, sensor hysteresis or increased blood flow in the forearm (as a result of gripping). This behavior was observed in all *relaxed* sensor data (*S1*–*S8*). In Figure 7B, the TAB readings correspond to gripping forces ranging between 20% and 60% of the maximum recorded force. In the 3D plot, a more normalized distribution is visible, in contrast to the histograms presented in Figures 3–6 which do not break down the gripping force. The visible diagonal indicates a linear correlation of the grip force with the TAB *S6* force readings. This happens despite the variability in the *relaxed* contact forces recorded across participants (Figure 7A) caused mainly by TAB fit and forearm adipose tissue content. This could be a result of the general increase in the physiological cross-sectional area (PCSA) of the forearm and *S6* location near the radius bone. However, it is also, more specifically, an indication of muscle activity in the proximity of the sensor.

### 3.2. Separability of Tactile Signatures

Following initial data analysis, statistical analysis was used to determine whether the four grip configurations of the hand while *relaxed* or *gripping* are statistically different and thus separable. That would provide some indications on the possible predictive algorithm that could be used to classify the data and statistical evidence of the tactile signatures mapping to the muscle synergies involved in each grip type. The data were split in eight groups; with the hand *relaxed* or *gripping* in the 4 positions shown in Figure 2. The separability of all these states is important for the development of predictive algorithms that are transparent and based on physiological cues. For the creation of a generalized model, that can perform well with all TAB users, it is paramount that there are similar patterns across the participants.

The two-sample Kormogolov-Smirnov (KS) test, run in Matlab using the *kstest2* function (MathWorks, 2018), was chosen to make the necessary comparisons as it does not assume Gaussian distribution. After testing each TAB sensor data normality, it was concluded that the data do not adhere to a Gaussian distribution (this is also evident in Figures 3–6). In this two-sample KS test, the hypothesis with regards to the distribution of a dataset is rejected by comparing the *p*-value with the significance level *Alpha* (default value of 0.05). *D*, the test statistic, is an indication of the “distance,” or difference, between the two samples' distributions when using the *kstest2* function. When it is 0, the data follow the exact same distribution as the specified one, the higher the value the greater the difference between the two data distributions.

No statistically significant similarity was found when analysing and comparing the individual sensor datasets for each grip type across participants. This was attributed to the differences in the forearm shapes and their adipose tissue-to-fat content ratio, as well as experimental errors and confounding variables. The latter includes the tightness of the TAB on each individual, the spacing of the TAB sensors and the surface area covered by the eight sensors in relation to the total circumference of the forearm (the larger the circumference the lower the coverage and thus the lower the sensing resolution). Nonetheless, statistically significant differences were found between the *relaxed* and *gripping* states across all 20 participants. The KS test statistic value, *D*, calculated for the *relaxed* state datasets across participants (for the individual grip types) was under 0.1. Therefore, 0.1 was set as a critical test value to determine statistically different distributions. Thus, any statistical difference between two data samples would need to be confirmed by both the null hypothesis and the critical test value.

#### 3.2.1. Finger Pose Signatures

The TAB data for the *relaxed* state were compared between the four hand grip configurations. Individual participant data were used with the two-sample KS test. The hypothesis tested was that the TAB sensor readings of different grip configurations belong to the same distribution. The test was run for each of eight TAB sensors and in each case the hypothesis was rejected in more than 99% of the data while the value of *D* always exceeded the critical value (0.1), with one exception. These results suggest that, even when no force is exerted on the gripping device, the position and cross-sectional area of the muscles that hold the joints at the four different hand grip configurations give rise to distinct tactile signatures. *D* values ranged from 0.14 to 0.999, indicating that the forearm undergoes greater changes in some areas than others while transitioning between the four hand configurations. However, this observation was not consistent amongst all participants.

Even in the *relaxed* state where the force differences are subtle the results agree with the grip analysis and muscle contractions. For example, between *Grip3* and *Grip4*, the ventral radial part of the forearm was expected to provide information to distinguish between the two (section 2.3.1). *S1* and *S2* would be the features to look out for, rather than the sensors at the ulnar part of the forearm (in particular *S6* which is in the proximity of the FDP). Looking at individual participant *S1*/*S2* data, the statistical distance (*D*) between the two distributions was over 0.5 while for *S6*, in most cases, it was under 0.2. However, the importance of these features was not clear when all participant data were considered. *D* values were lower than 0.2 in most cases for the force distributions or *S1* or *S2* at the two different hand configurations. Furthermore, the tactile signatures of *Grip1* and *Grip4* in the *relaxed* state were the most similar despite the two hand configurations being very different.

Overall, the force density distributions on the TAB sensors for each hand configuration and across participants were different. This is an indication that the creation of a generalized model is probably unfeasible with the current data. Nonetheless, the tactile signature differences of the four hand poses in the *relaxed* state were found to be more significant than these variations across participants, as the KS statistic value, *D*, was found to be much higher. The sensors with the tighter distributions, and higher *D* value would be the most useful features for classification of the four different hand grip configurations. However, given the variability of the tactile signatures across the participants, a combination of all TAB sensor responses would be needed to determine the hand pose.

#### 3.2.2. Relaxed vs. Gripping

**Figure 8:**
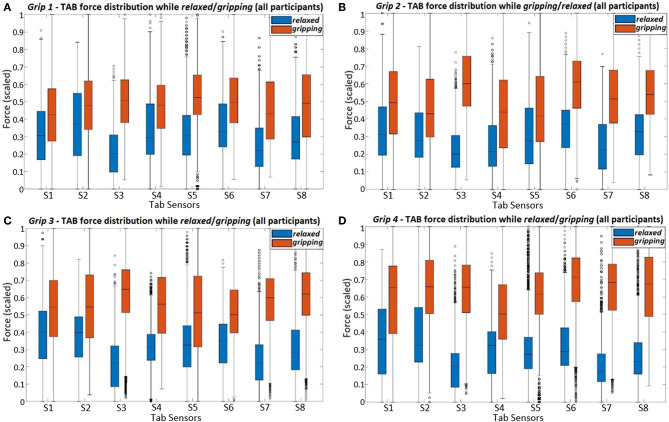
The figure presents the force readings of each TAB sensor (using all participant data) for the four different hand configurations; **(A)** *Grip1*, **(B)** *Grip2*, **(C)** *Grip3*, and **(D)** *Grip4*.

How can the TAB differentiate a hand *gripping* from being *relaxed*? What is the TAB sensors' response to different grips? Are these features common across participants? The 2-sample KS test was used to compare the *relaxed* and *gripping* sensor data. Figure 8 presents the force readings of all participant data for each of the four grip types. *S3* records distinct force changes across all grip types. This sensor is not near the digit flexors which are responsible for gripping but is in the proximity of the radial bone where there is a low adipose tissue content forming a more rigid contact surface. As the large digit flexor muscles contract, their stiffness and PCSA increase (PCSA being proportional to muscle force), in turn increasing the forearm's cross sectional area. This generates higher normal and shear forces on the arm brace, the former being monitored by the TAB sensors. These forces are transmitted more efficiently on the brace by the more rigid parts of the forearm. Thus, *S3* can be a consistent general indicator of *gripping* but possibly not as good of a feature for differentiating grips.

The force distributions at *S8, S7*, and *S6* agree with the grip analysis (section 2.3.1). The combination of these three sensors provide good features for predicting the grip type. When different parts of the FDP and FDS muscles engage during *gripping* there is a clear separability in the recorded force distributions. As expected (Table 2), the *S6* data have the best separability during *Grip4* (Figure 2D). This is evident in Figure 8D and the higher KS statistic values, *D*, calculated when the little and ring fingers engage. Despite the broad force density distributions across participants, there is a clear separability of the *relaxed* and *gripping* states in the *S7* and *S8* data. These two, as expected, are better predictors of *Grip2* and *Grip3* than *S6*, due to their proximity to the corresponding muscles. For *Grip1*, what was surprising was the similarity of the *S1 relaxed* and *gripping* distributions.The results of the remaining sensors are in agreement with the expected outcomes presented in Table 2.

**Figure 9:**
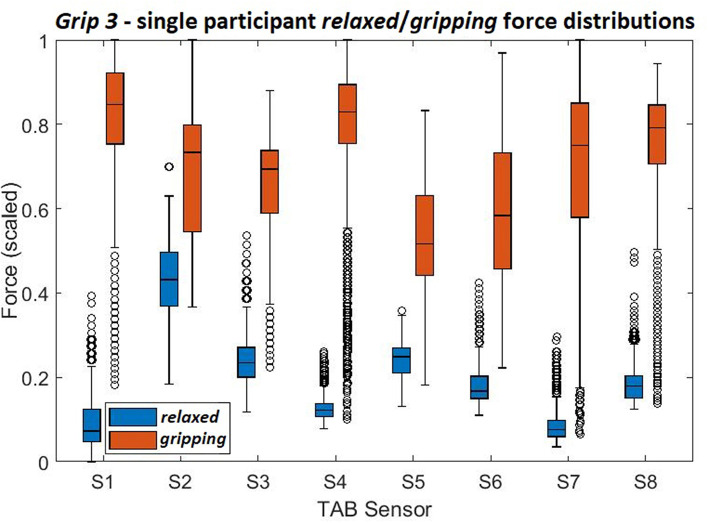
The force density distributions, *relaxed* and *gripping*, from a single participant's data when performing *Grip3*.

Comparisons of Figure 9 and Figure 8C show how the separability of the *Grip3* data is much clearer in a single participant's data. The force variance observed on *S4* between the *gripping* and *relaxed* states is probably due to the lower adipose tissue content in that region of the forearm. The same applied to *S1* but despite the sensor's proximity to the flexor muscles this was not the case for all participants.

Overall, the results indicate that most of the sensors show a statistically significant difference between the states which suggests that a data-driven, linear algorithm may prove sufficient at identifying these states, despite the broad force distributions. Using the overall tactile signature of the forearm for motion intent recognition would work better than just focusing on particular regions. Also, as evident by the state separation in Figure 9 a personalized system would work better than a generalized one.

### 3.3. Principal Component Analysis (PCA)

Given the evidence of linear separability in the data, PCA was performed, using the IBM SPSS software (IBM, 2019), to determine the extent to which the TAB data variability of each grip type across states can be expressed by linear components. Eight different components that describe the variance in the data were produced. Using the Keiser criterion only the ones with an eigenvalue greater than 1 were considered. The *Varimax* method was used and a maximum of 200 iterations were allowed for convergence. It is recommended that the components chosen describe 70–80% of the data variance. However, the principal components (PCs) explain 60.5, 68.7, 65.7, and 77.4% of the variance of *Grip1-4*, respectively. The lower values are due to the physiological differences between the participants. The results are in line with the grip analysis (section 2.3.1, Table 2). The PCs' highest correlations are observed with the sensors placed in the proximity of the digit flexors, *S6, S7* and *S8*, and *S3*. *S3* was found to be a good indicator of *gripping* but not of differentiating between the grip types (section 3.2.2).

### 3.4. Tactile Signatures—Expectations vs. Results

During multiple participant experiments, the forearm tactile signature on the TAB mostly aligns with the muscle physiology and muscle synergies. Some observed incoherence in the data suggests that personalization of the TAB would greatly improve its accuracy. The variability between participants that affects the baseline readings and accentuates certain features over others can be attributed to:

- Forearm shape – causes variations of the baseline readings
- TAB fit - tighter fit elevates the baseline readings, especially in the proximity of the ulna and radius, a loose fit may not detect muscle activity
- Adipose tissue content - lowers the baseline readings and dampens the output
- Forearm strength/muscle mass raises baseline and makes the output more prominent
- Forearm circumference affects the distance between the sensors and their proximity to individual muscles.

The tactile signatures of individual forearm muscle contractions can be used to create a map of each muscle contraction from the TAB response. This can be implemented in a motion intent recognition system to actuate an exoskeleton, instead of (or in combination with) training a machine learning model on particular motions. The advantage of this approach is that even unknown sensory inputs will provide a reliable control input increasing the robustness of the exoskeleton control.

## 4. Tab State Prediction

Machine learning techniques were employed to create generalized models from the TAB data recorded in the experiments. To start with, a model was created for each grip type, individually, to predict whether the hand is *gripping* or *relaxed* (binary classification). All grip types' *relaxed* data were then grouped together to predict which of the four grip types is being performed when the hand is *gripping* (5 classes). Following that, the algorithms were ran on all *relaxed* state data to create a model that discerns between the four grip configurations and the subtle differences between the four relaxed hand poses (4 classes). Finally, all TAB data were used to classify the four *relaxed* and four *gripping* states (8 classes).

### 4.1. Features

Engineered features were created and ranked to improve the performance of the predictive algorithms for individual grip types as well as for the overall dataset. Both the temporal and spatial tactile patterns can provide characteristics of different hand movements despite the differences in the TAB response for each participant. It is, therefore, important to take into consideration the history of each sensor's readings (considering the change of force within about 0.5 s) as well as the spatial features which were generated from the differences between each of the TAB sensor force readings. The differences of the sensor readings could provide information on antagonistic muscle pairs or combinations of muscle contractions. Concatenating these features with the TAB sensor readings, a total of 44 features were established. The first eight are the TAB sensor readings [*S*_1_, …,*S*_8_], 9–16 their derivatives [*dS*_1_, …,*dS*_8_] and features 17-44 are the force reading differences between each pair of sensors; i.e., [*S*_1_ − *S*_2_, *S*_1_ − *S*_3_, …,*S*_7_ − *S*_8_].

The most important of these features were extracted for grip classification. Two approaches were taken to determine feature importance. In the first, the features were ranked using the Random Forests (RF) algorithm where the first 10 features were chosen for each classification scenario. The second approach transformed the engineered features with the principal components established using PCA. All individual sensor data, *S1*- *S8*, and some spatial features were found to be important. For *Grip1* these included [*S*_7_−*S*_3_, *S*_8_−*S*_2_], for *Grip2* [*S*_8_−*S*_3_, *S*_5_−*S*_3_], for *Grip3* [*S*_6_ − *S*_3_, *S*_7_ − *S*_6_], and for *Grip4* [*S*_4_ − *S*_3_, *S*_8_ − *S*_4_]. The temporal features, on the other hand, did not provide any useful information. The results are in line with our initial analysis as the spatial data that featured high on the importance list for individual grips mainly involved *S3*. *S6, S7*, and *S8* were also key sensors in these spatial data. As seen earlier, *S3* and *S6* are good indicators of *gripping* due to the high variability in their data distributions during gripping. *S8, S7*, and *S6* data are useful features in distinguishing between the four grip types. A number of machine learning techniques were employed to create models and make state predictions. The models were trained on 80% of the data and tested on the remaining 20% of participants' data.

### 4.2. Learning the Tactile Features

Using the most important features calculated by the RF algorithm and the PCA-transformed data, supervised learning algorithms were implemented with a 10-fold cross-validation. There are multiple learning algorithms that can be used for classification purposes, both parametric and non-parametric. The k-nearest-neighbor (kNN) (Akhlaghi et al., 2016), logistic regression, random forests (RF) and support vector machines (SVM) (Wolf et al., 2013) were all implemented using the python scikit-learn library.

The prediction accuracy was higher when using the chosen engineered features in comparison with using solely the *S1*−*S8* readings. Using all engineered features instead of only the ones found to be of importance has no significant effect on the results (h = 0). Being selective with the features decreases the computational time without compromising the accuracy. The results indicated that although the accuracy differences between the algorithms were not significant, the SVM had the best overall performance. When attempting to distinguish between all grip types and *gripping*/*relaxed*, a total of 8 classes, only about a quarter of the data were correctly classified. The kNN algorithm was not able to capture the complexity of the features as well as an ensemble method like RF. The SVM models, which used a Gaussian kernel, also resulted in higher prediction accuracy than kNNs and performed marginally better than RF in determining the state of the individual grips and classifying all grips and positions. The prediction accuracy using the principal components of the TAB data was the highest in the classification of *Grip3*. This could be due to the fact that the ratio of the maximum force allowed during the experiment, to the maximum that can be generated using that grip type is the highest for *Grip3* (as evident in Table 1).

The high standard deviation of accuracy of all machine learning algorithms demonstrates a great variation of the TAB response within the participant pool. The training data and validation data prediction accuracies differed and only about a quarter of the data were correctly classified. Using the default parameters of the scikit-learn library, the SVM model generated with the features chosen using RF performed better than the one with the PCA transformed features. An exhaustive search over the *gamma* and *C* parameter values of the SVM was performed using grid search. This revealed that the model performs best when gamma is 1.0 and C is 1. Using the optimal parameter values, the SVM model's performance improved but it still failed to reach the desired accuracy as evident in Table 3.

**Table 3:** The classification accuracies (mean and standard deviation) achieved using the SVM models trained on the PCA transformed features and the RF selected features, with a 10-fold cross validation.

**Feature acquisition**	**PCA**	**RF**
Grip1	77.10 ± 9.01	81.42 ± 10.03
Grip2	84.78 ± 7.4	85.76 ± 8.70
Grip3	89.30 ± 6.06	93.43 ± 5.03
Grip4	83.54 ± 11.27	88.44 ± 8.55
All grips, all positions	22.37 ± 9.86	27.14 ± 9.26
All grips and all positions	54.06 ± 11.35	56.13 ± 10.38
All positions (relaxed)	22.37 ± 9.86	23.97 ± 7.03

The model did not perform well when tested on the data of the 4 “unseen” participants (20% of the data). This indicated that the variability across the population is not adequately captured with such a small number of participants to allow for generalization. The model was then trained on partial data from all participants. The data were shuffled prior to being split into the hold-out groups which meant that the training sample would most probably include some data from all 20 participants. This raised the model performance, a seen in Table 4. The most notable improvements were made with 8-way classification (4 *gripping* and 4 *relaxed* states) and on classifying the 4 relaxed hand configurations where the differences are subtle. The accuracy for both increased to over 95%. This indicates that using these learning algorithms the model would need to be personalized to the user.

**Table 4:** Training on all participants' data.

	**Classification accuracy (%)**	
**Features**	**PC**	**RF**
Grip1	96.19 ± 0.20	97.06 ± 0.11
Grip2	97.61 ± 0.16	98.07 ± 0.17
Grip3	98.17 ± 0.10	98.96 ± 0.11
Grip4	99.10 ± 0.10	99.44 ± 0.06
All grips, all positions	97.60 ± 0.04	93.75 ± 0.15
All positions (relaxed)	95.89 ± 0.26	96.75 ± 0.12

*The classification accuracies (mean and standard deviation) achieved using the SVM models trained on the PCA transformed features and the RF selected features, with a 10-fold cross validation*.

**Figure 10:**
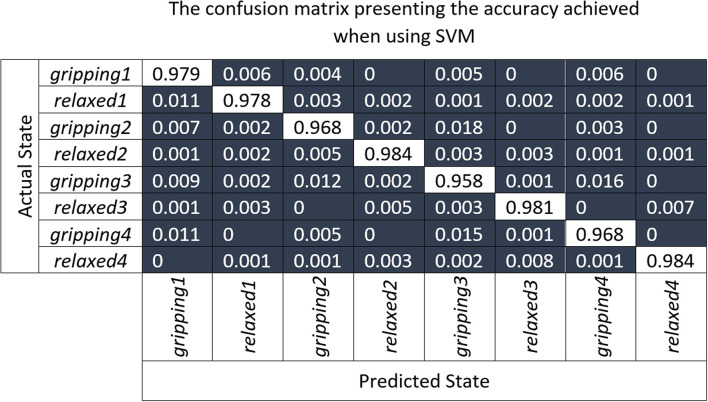
The confusion matrix presenting results of the 10-fold cross-validation of the SVM model which was trained using the principal component transformed features of partial unseen data from all participants.

The confusion matrix (Figure 10), shows the results of the 10-fold cross-validation of the SVM model which was trained using the principal component transformed features of partial unseen data from all participants. The labels *relaxed1*/*gripping1* represent the *relaxed*/*gripping* states of the *Grip1* hand configuration and the same applies for the other three grip types. It can be observed that the highest false negatives in the “one-vs.-rest” (training a single classifier per class) SVM approach were found when trying to distinguish between:

- *Grip3* and *Grip4* which use all fingers; 1.6% of *gripping3* was incorrectly classified as *gripping4* and 1.5% of *gripping4* as *gripping3*
- *Grip2* and *Grip3* with similar trapezoid shapes; 1.8% of *gripping2* was incorrectly classified as *gripping3* and 1.2% of *gripping3* as *gripping2*.

Table 1 shows that the ring and little finger do not contribute as much force as the index or middle finger during *Grip3* which contributes to its miss-classification as *Grip2*. Overall, *Grip3* has the highest false positive rate with 4.7%. *Relaxed4* is the one with the lowest false positive rate, with 0.9%. The relaxed states were, overall, classified slightly better than the gripping states possibly due to some difference in muscle engagement between the participants. In summary, the highest miss-classification of a state is for *gripping3*, 4.2%, the lowest for *relaxed4* and *relaxed2*, 1.6%, with an average of 2.5% across all states.

The number of participants involved in this study was not sufficient to allow creation of a generalizable intent recognition model that can perform accurately (e.g., 90%) on the unseen participants. Nonetheless, the TAB system performance achieved an average classification accuracy of 99.96 ± 0.08% which is comparable to the state-of-the-art motion intent recognition systems not tested on weak muscle activations (Wolf et al., 2013; Xiao et al., 2014; Li et al., 2017).

### 4.3. TAB vs. Myo Armband

#### 4.3.1. Comparative Study

The two sensorised armbands, the TAB and the Myo, offer two different means of detecting muscle contractions. The first uses interaction forces on the forearm while the other detects electrical signals that reach the skin surface. The TAB experiments were replicated with the same participants using Myo armband (Thalmic Labs, 2015c). The Thalmic Labs' Myo Gesture Control Armband (Thalmic Labs, 2015c) features an Inertial Measurement Unit (IMU) and eight sets of sEMG. These are accessed using the Windows SDK provided. The Myo was chosen for comparison with the TAB for its ease of use, adaptability to various forearm sizes and the number of measurement points (8) which is the same as in the TAB. The sEMG data were sampled at around 100 Hz. Positioning of the Myo EMG sensors on the forearm was done according to the manufacturers instructions (Thalmic Labs, 2015a).

#### 4.3.2. sEMG: Features and State Prediction

The same 44 engineered features as earlier were produced for the Myo data. The temporal features included voltage changes over 0.5 s windows, as in Wolf et al. (2013). The RF algorithm was used to rank the features and the 10 most important were chosen. The temporal features ranked high in contrast to the TAB where they ranked the lowest. In both, cases, however, the radial part of the forearm provided key features. All participant data were shuffled and a 10-fold cross-validation was performed using the kNN, SVM and RF learning algorithms. The best performance was achieved with the RF model (Table 5). The highest prediction accuracy with the Myo data was achieved for *Grip4*, where the force limit set for the experiment was the highest amongst the grip types (Table 1). Higher forces engage more muscle fibers, creating a higher potential difference and therefore stronger signals to be detected by sEMG sensors. Overall,the classification accuracy for individual grip types seems to be dependent on the grip force being used since *Grip*1_*accuracy*_ < *Grip*2_*accuracy*_ < *Grip*3_*accuracy*_ < *Grip*4_*accuracy*_.

**Table 5:** The classification accuracy achieved using RF on the Myo EMG (10-fold cross-validation) data.

	**Random forest**
Grip1	65.12 ± 6.92
Grip2	69.23 ± 10.00
Grip3	73.73 ± 11.19
Grip4	78.81 ± 9.11
All grips, all positions	26.01 ± 2.956
All grips and relaxed	40.21 ± 11.22
All positions (relaxed)	40.56 ± 4.07

#### 4.3.3. Performance: TAB vs. Myo

The tactile and sEMG data have different types of key features. Temporal features were important when classifying sEMG data, which confirmed results from Wolf et al. (2013). With the TAB tactile data the most prominent features were the eight raw sensor readings followed by a number of spatial features. The PCA transformed features performed at similar levels as the RF selected features. The best performing algorithm was the SVM. It is worth noting that more complex and computationally expensive algorithms such as a recurrent neural network (LSTM) were also implemented with similar accuracy to SVM. SVMs are commonly used in this type of application, with examples like NASA's Biosleeve project (Wolf et al., 2013) and McIntosh's SVM implementation in a multimodal sensing (sEMG and FSR) system (McIntosh et al., 2016).

Myo EMG data classification accuracy is highly dependent on the gripping force which is not the case with the TAB data classification. The TAB system confusion matrix was indicative of its high performance (over 90%). The corresponding Myo system matrix indicated its inability to correctly identify the data. In the classification of the four *relaxed* hand configurations, the majority of the data were classified as *Grip1*. Only 0.4% of the *relaxed* power grip is classified correctly. The weak electrical signals generated by low gripping forces seemed unable to reach the skin surface. Cross-talk may be another reason for this low-accuracy performance.

The results show that a motion intent recognition system that uses tactile sensing can achieve high classification accuracy (>90%) when personalized. Distinguishing between four different static hand positions and 4 grips, in the same positions (a total of 8 classes), >99% classification accuracy can be achieved. This is comparable to the state-of-the-art systems that use sEMG, such as NASA's BioSleeve (Wolf et al., 2013). There were not enough participants in this study to create a generalized TAB system. The need for personalization is not unique to the TAB or the general force/pressure approach when a small number of participants is used. This has been recognized in similar studies that achieved accuracy as high (Wolf et al., 2013; McIntosh et al., 2016). Under the same conditions the Myo sEMG data classification did not perform as well.The Myo data trained models were unable to distinguish between the different classes. A more sophisticated sEMG system may have been more sensitive to the low-strength motions used and thus more capable of making distinctions between the states.

This study proves that the TAB can achieve as high hand motion classification accuracy as the state-of-the-art sEMG systems (Phillips and Craelius, 2017). A more extensive study with a broader range of motions would elicit the limitations of the TAB and confirm the results obtained in this study. More testing is required to identify the extent to which adipose tissue content affects the TAB performance. Moreover, the sensor placement with respect to the distance from the proximal elbow joint and the ability to monitor particular muscles should be explored. Algorithms such as SVMs have the potential to correctly classify different data from the same participants. However, if a higher accuracy is needed for a generalized model then a much larger population is required in order to capture the distribution of forearm sizes, shapes and adipose tissue and muscle content. Especially since the resolution of the tactile signature was very much dependent on the forearm circumference as the sensing area was always constant.

## 5. Conclusions and Further Work

The integration of the TAB in rehabilitative and assistive devices is well justified through the experiments performed that emulated low strength arm/hand conditions. It has been shown that the forearm tactile signatures can be mapped to particular muscle contractions. These could be used as a control input to a wearable device the ability to respond to all sensory inputs appropriately without having to “learn” motion primitives. Achieving such transparency in the system would improve safety of wearable autonomous devices. The TAB was able to detect the tactile signature differences under such conditions and achieve higher classification accuracies than the sEMG of the Myo armband. The personalized model trained on partial PC-transformed data from all participants achieved an accuracy of 95.9% when distinguishing between the four *relaxed* hand positions. The 8-state (four *relaxed* and four *gripping*) classification accuracy was 97.6%.

In this study the mechanical cues that arise as a result of weak muscle contractions were captured by recording the normal forces generated on an arm brace. The use of higher sensitivity sensors, an array of which would cover the entirety of the TAB circumference could improve the prediction accuracy and the resolution would no longer be dependent on the forearm circumference. This could potentially also provide better data for the generalization of the system. Furthermore, incorporating sheer force sensing in addition to the normal force monitoring could further improve the TAB performance. To conclude, this study with the TAB system has shown that force myography is a promising motion intent recognition technique that could be potentially useful for upper-limb rehabilitation devices providing the transparency required for inherent safety.

## Data Availability Statement

The datasets generated for this study are available on request to the corresponding author.

## Ethics Statement

This study was carried out in accordance with the recommendations of the Faculty Research Ethics Committee at the University of the West of England (UWE REC REF No: FET.18.02.028) with written informed consent from all subjects. All subjects gave written informed consent in accordance with the Declaration of Helsinki. The protocol was approved by the Faculty Research Ethics Committee.

## Author Contributions

TS conceived the presented idea, preformed the experiments and the data analysis, and interpretation under the supervision of SD, GC, and TA. TS drafted the article which SD and GC discussed and commented on.

### Conflict of Interest

The authors declare that the research was conducted in the absence of any commercial or financial relationships that could be construed as a potential conflict of interest.

## Footnotes

^1^The maximum strength and the individual fingers' force contributions to *Grip3*, a prismatic 2 finger grip, grip No.8 in NCD Risk Factor Collaboration (NCD-RisC), were not found hence the tripod grip, grip No.14, values were used from Kinoshita's study (Kinoshita et al., 1996).
