# Supplementary material for: Tactile Signatures and Hand Motion Intent Recognition for Wearable Assistive Devices
Source: Front Robot AI. 2019 Nov 21;6:124. doi: 10.3389/frobt.2019.00124 (PMC7805773; doi:10.3389/frobt.2019.00124)
Supplement: Supplementary file 1 [file Table_1.pdf]

Appendix - Supplementary Material

Principal Component Analysis of the Grips:

Table 1. A tabulation of the loadings of the principal components (with an eigenvalue higher than 1.0) of the data for each grip type.

	Grip1		**Grip2**	
**PC**	**Var (%)**	**Highest Correlation**	**Var (%)**	**Highest Correlation**
**PC1**	46.0	*S8/S3/S6*	49.57	*S3/S6/S8/S7*
**PC2**	14.6	*S5*	19.13	*S1*
**Total (%)**	60.5		68.70	

	**Grip3**		**Grip4**	
**PC**	**Var (%)**	**Highest Correlation**	**Var (%)**	**Highest Correlation**
**PC1**	49.6	S3/S7/S8	64.5	S3/S8/S7
**PC2**	16.1	S5	12.8	S1/S4
**Total (%)**	65.7		77.4	
