# Supplementary material for: Tactile Signatures and Hand Motion Intent Recognition for Wearable Assistive Devices
Source: Front Robot AI. 2019 Nov 21;6:124. doi: 10.3389/frobt.2019.00124 (PMC7805773; doi:10.3389/frobt.2019.00124)
Supplement: Supplementary file 2 [file Image_1.pdf]

TAB sensors' Force Reading Distributions:

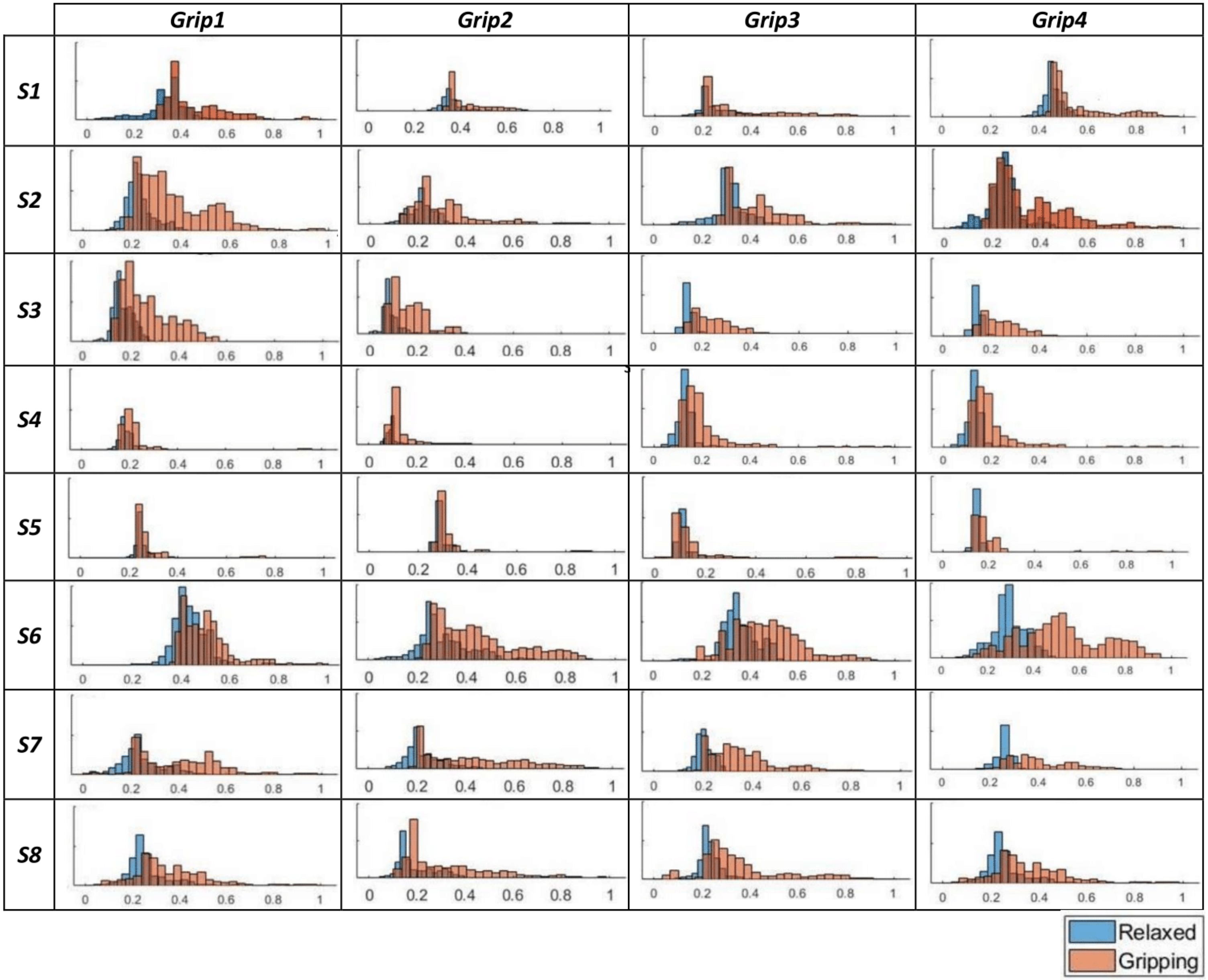

Figure 1. This matrix makes comparisons of the force distributions across grips as well as across sensors for individual grip types (with all participant data). The forces recorded by each sensor while the hand is *relaxed* are presented in blue and when *gripping* in orange.
